# A Sensitive Plasma Insulin Immunoassay to Establish the Diagnosis of Congenital Hyperinsulinism

**DOI:** 10.3389/fendo.2020.614993

**Published:** 2021-02-19

**Authors:** Julie Siersbæk, Annette Rønholt Larsen, Mads Nybo, Henrik Thybo Christesen

**Affiliations:** ^1^Hans Christian Andersen Children’s Hospital, Odense University Hospital, Odense, Denmark; ^2^Department of Clinical Research, Faculty of Health Sciences, University of Southern Denmark, Odense, Denmark; ^3^Department of Clinical Biochemistry and Pharmacology, Odense University Hospital, Odense, Denmark; ^4^Odense Pancreas Center (OPAC), Odense University Hospital, Odense, Denmark

**Keywords:** hypoglycemia, congenital hyperinsulinism, children, diagnostic performance, immunoassays

## Abstract

**Background:**

The diagnosis of congenital hyperinsulinism (CHI) may be hampered by a plasma (p-) insulin detection limit of 12–18 pmol/L (2–3 mU/L).

**Objective:**

To evaluate the diagnostic performance of a sensitive insulin immunoassay and to find the optimal p-insulin cut-off for the diagnosis of CHI.

**Methods:**

Diagnostic fasting tests, performed without medication or i.v.-glucose, were investigated in children with a clinical diagnosis of CHI, or idiopathic ketotic hypoglycemia (IKH). The CHI diagnosis was either clinical or by the alternative, p-insulin-free criteria; hypoglycemia plus disease-causing genetic mutations and/or CHI-compatible pancreatic histopathology. We included diagnostic p-insulin samples with simultaneous p-glucose <3.2 mmol/L and used a sensitive insulin assay (Cobas e411 immunoassay analyzer; lower detection limit 1.2 pmol/L; normal range 15.1–147.1 pmol/L). Receiver operating characteristics area under the curve (ROC AUC) values and optimal cut-offs were analyzed for the performance of p-insulin to diagnose CHI.

**Results:**

In 61 CHI patients, the median (range) p-insulin was 76.5 (17–644) pmol/L compared to 1.5 (1.5–7.7) pmol/L in IKH patients (n=15). The ROC AUC was 1.0 for the diagnosis of CHI defined both by the clinical diagnosis (n=61) and by alternative criteria (n=57). The optimal p-insulin cut-offs were 12.3 pmol/L, and 10.6 pmol/L, at p-glucose <3.2 mmol/L (n=61), and <3.0 mmol/L (n=49), respectively.

**Conclusions:**

The sensitive insulin assay performed excellent in diagnosing CHI with optimal p-insulin cut-offs at 12.3 pmol/L (2.0 mU/L), and 10.6 pmol/L (1.8 mU/L), at p-glucose <3.2 mmol/L, and <3.0 mmol/L, respectively. A sensitive insulin assay may serve to simplify the diagnosis of CHI.

## Introduction

Hyperinsulinemic hypoglycemia (HH) is a heterogeneous condition with dysregulated secretion of insulin from the β-cells during hypoglycemia. The incidence of the congenital variant congenital hyperinsulinism (CHI) is approximately 1:30,000–50,000 in populations without founder mutations (1, 2). Although the condition is rare, it is the most common cause of severe hypoglycemia in neonates and infants.

The clinical manifestations of HH vary from unspecific hypoglycemic symptoms to loss of consciousness, seizures and coma (3–5). Neurodevelopmental impairment is seen in 25%–50% of patients with CHI and 13%–25% of the patients develop epilepsy, indicating insufficient prompt treatment of the hypoglycemia (2, 5–7). Mutations in at least 12 different genes (*ABCC8, KCNJ11, GLUD1, GCK, HADH, SLC16A1, UCP2, HNF4A, HNF1A, HK1, PGM1*, and *PMM2)* have been identified in non-syndromic HH. Mutations are by far most common in the β-cell K_ATP_-channel genes *ABCC8* and *KCNJ11*, where loss of function leads to CHI while gain of function leads to monogenic diabetes (2, 8–10). The main goal in the treatment of CHI is to maintain a blood glucose level >3.5 mmol/L (>63 mg/dl) (11), or >3.9 mmol/L (>70 mg/dl) (2), to avoid brain damage. Treatment modalities include i.v. glucose and glucagon followed by, primarily, diazoxide, octreotide and long-acting somatostatin analogues. Restricted pancreatic surgery is indicated in the focal type, whereas subtotal pancreatectomy may be the ultimate choice in diffuse CHI if medical therapy fails (12).

The biochemical diagnosis of HH may be troublesome. Beyond 3 days of age, hypoglycemia may be defined as p-glucose <2.8 mmol/L (50 mg/dl), <3.0 mmol/L (55 mg/dl) or <3.3 mmol/L (60 mg/dl), although no single cut-off can define hypoglycemia with respect to the risk of brain damage (1, 12–14). As the key element, HH is defined as simultaneous hypoglycemia and detectable serum or plasma (p-) insulin, i.e. ≥2–3 mU/L depending on the assay used (7, 15). As the insulin concentration in up to 20% of CHI patients may be undetectable, supporting evidence is often necessary to establish the diagnosis. This includes hypoketonemia, low fatty acids, a high glucose infusion demand (>8–10 mg/kg/min) to ensure normoglycemia, and an increased response to i.m. glucagon (10, 15–17).

In other HH disorders, e.g. insulinoma, supporting diagnostic evidence is the insulin-glucose ratio, or the amended insulin-glucose ratio, where 1.7 mmol/L is subtracted from the glucose value (18, 19).

Ketotic hypoglycemia may be idiopathic (IKH) when known causes to hypoglycemia are excluded, such as CHI, glycogen storage diseases (GSD), other metabolic diseases, deficiency of growth hormone, and adrenal insufficiency. IKH is usually less severe, but more common than CHI in infancy. In IKH, which typically presents around 6 months of age, p-insulin is adequately suppressed during hypoglycemia (20, 21). A sensitive assay may, however, show precise p-insulin concentrations at otherwise undetectable concentrations in IKH patients.

We aimed to utilize our sensitive immunoassay to determine the optimal cut-off for p-insulin for the diagnosis of CHI.

## Patients and Methods

### Design and Setting

In a retrospective observational design, we investigated laboratory values and clinical data in patients with CHI or IKH, admitted to the International Hyperinsulinism and Hypoglycemia Centre, Hans Christian Andersen Children’s Hospital at Odense University Hospital, Denmark. The study complied with the STARD reporting guidelines for diagnostic studies (22).

### Patients

Laboratory data from all patients aged 0–15 years with measurement of p-insulin in the period February 2013-August 2019 were retrieved from the hospital’s laboratory information system BCC (CGI, Kolding, Denmark). Medical records were checked for the presence of hypoglycemia and related diagnoses. As our department used 3.2 mmol/L as a cut-off for diagnosing hypoglycemia, samples with a simultaneous p-glucose ≥3.2 mmol/L at the time of measurement were excluded, no matter the diagnose of the patient. Exclusion criteria in samples with p-glucose <3.2 mmol/L were other diagnoses than CHI or IKH, e.g. diabetes, KH of known cause and uncertain diagnoses, and age less than 1 week, corrected for gestational age at time of blood sampling.

The retrospectively validated diagnosis of IKH required hypoglycemia with simultaneous blood ketones >1.5 mmol/L and exclusion of known metabolic and hormonal diseases with hypoglycemia. The clinical diagnosis of CHI was also retrospectively validated as inappropriately elevated p-insulin during hypoglycemia in samples from our laboratory and eventual supporting criteria (14, 15). Furthermore, an alternative diagnosis of CHI without the use of p-insulin was retrospectively established with the following criteria: hypoketotic hypoglycemia plus disease-causing genetic mutations and/or diffuse, focal, or atypical pancreatic histopathology compatible with CHI in surgery-treated patients.

A further exclusion criterion was blood samples obtained during ongoing medication for hypoglycemia. Samples from patients with suspicion of residual medication concentrations due to recent discontinuation were, however, not excluded if p-insulin was >55 pmol/L during hypoglycemia, as clearly high values were not anticipated to affect the determination of p-insulin cut-off values in the diagnosis of CHI.

**Figure 1:**
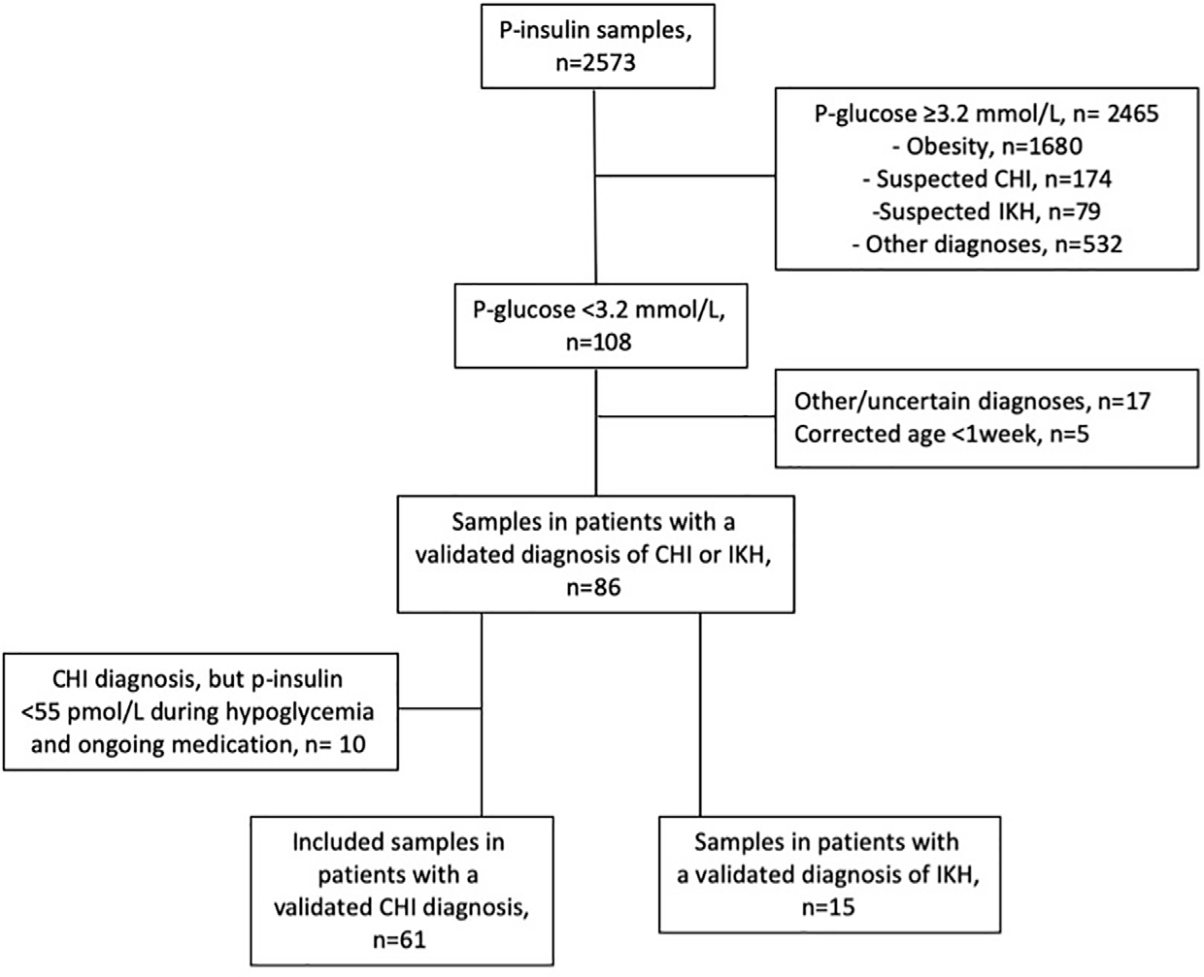
Patient inclusion flowchart. CHI, congenital hyperinsulinism; IKH, idiopathic ketotic hypoglycemia; p, plasma.

A patient inclusion flowchart is presented in **Figure 1**. In the period studied, a total of 74 CHI and 59 IKH patients were seen at our institution. Of these, 25 CHI patients and 9 IKH patients had blood sample p-insulin with a simultaneous p-glucose that did not meet our inclusion criteria. By cross-checking with a diagnosis-based database on patients with hypoglycemia, 0 CHI and 36 IKH patients did not have p-insulin measured with simultaneous central laboratory p-glucose at our institution. In addition, seven patients had GSD IXa (ketotic), two had carnitine palmitoyl transferase 1 deficiency, and one had GSD Ia (non-ketotic). Patients with other hypoglycemia-related diseases, but no recorded hypoglycemia diagnosis, were not retrieved by our searches.

For the finally included patients, the following data were obtained from the medical records: the retrospectively validated diagnosis, age at time of blood sampling, gender, nationality, genetic analyses, histopathological analysis, p-glucose, p-insulin, p-C-peptide, p-proinsulin, blood ketones, fasting duration prior to blood sampling and medical treatment.

### Laboratory Analyses

Since February 2013, p-insulin has been measured at our institution with a sensitive assay (Cobas e411 immunoassay analyzer, Roche^®^) with a limit of detection (LoD) of 1.18 pmol/L (0.2 mU/L), a limit of quantification (LoQ) of 1.7 pmol/L, and a normal range for fasting adults of 15.1-147.1 pmol/L. According to the manufacturer, the coefficient of variation is 1.9-2.0%, when tested for repeatability. The assay uses a conversion factor of 6.945 when converting insulin values from mU/L to pmol/L, which disagrees with the biological activity of insulin which is 6.00 nmol/IU (23, 24). To correct for this common mistake, we subtracted ≈15% by multiplying all p-insulin concentrations reported in pmol/L with 6.00/6.945, including the normal reference range. Uncorrected data were given for ease of comparison.

P-proinsulin and p-C-peptide were also measured with Cobas e411; p-proinsulin with a LoQ of 2 pmol/L and a normal range of 2–23 pmol/L for fasting adults, p-C-peptide with a LoQ of 1 pmol/L and a normal range of 400–1600 pmol/L for fasting adults. Glucose was measured on plasma from venous blood samples with Cobas 8000 hexokinase assay analyzer (Roche^®^) with a LoD and LoQ of 0.11 mmol/L and a normal range of 2.5–11 mmol/L for fasting adults. All blood samples were tested for hemolysis interference. Bedside glucose values, or continuous glucose monitoring values, were not used in the present study.

### Ethics

The Region of Southern Denmark, Protocol no. 19/7744, and the Danish Patient Safety Authority, Protocol no. 3-3013-2884/1, approved the study protocol. Ethic Committee approval was not relevant according to national regulations, as the study was observational only.

### Statistics

In descriptive statistics, numerical variables were expressed by median and range, while categorical variables were expressed in number and percentage. Corresponding p-values were calculated by Wilcoxon rank-sum (Mann-Whitney) for numerical variables. Pearson’s chi^2^-test was performed for categorical variables. Level of significance was p<0.05.

Ten samples from patients with IKH had p-insulin <1.7 pmol/L (LoQ). Since values were not expected to be normally distributed, these samples were arbitrarily assigned a concentration of 1.5 pmol/L for statistical handling. Likewise, p-proinsulin concentrations <2 pmol/L (n=12, IKH patients) were arbitrarily set to 1.5 pmol/L.

In order to determine the optimal cut-off for p-insulin, we performed receiver operating characteristics (ROC) curves with determination of area under the curve (AUC). The concordance probability method by Liu was used to define the optimal cut-off as the point that maximizes the product of sensitivity and specificity (25). The 95% confidence intervals were calculated using bootstrapping and 1,000 replications. All statistical analyses were performed using STATA 16 (StataCorp, College Station, Texas).

All included blood samples from the finally included patients were analyzed in the main analysis. To account for potential selection bias caused by oversampling, a sensitivity analysis was performed where only one sample per patient was included. The highest, or the first, insulin value was included, respectively.

As one single p-glucose cut-off for hypoglycemia in children cannot be set, an additional analysis was performed on the subgroup of blood samples with a p-glucose <3.0 mmol/L. Furthermore, analyses were performed on the subgroup of CHI patients who could be given an alternative diagnosis of CHI without the use of p-insulin as a diagnostic criterion.

The insulin-glucose ratio and the amended insulin-glucose ratio [p-insulin/(p-glucose - 1.7 mmol/L)] were calculated to evaluate the positive predictive value (PPV) of these supporting diagnostic parameters in CHI. Normative data are ≤32.2 pmol/mmol, and ≤53.6 pmol/mmol, respectively (18, 19). We calculated our ratios both without and with the correction factor 6.00/6.945 for robustness.

## Results

### Patients and Blood Samples

A total of 61 blood samples from patients with CHI were obtained from 49 patients. Oversampling occurred for eleven patients who had two samples and one patient who had three samples eligible for analyses. Ninety percent of the included patients with CHI were referred from other countries, **Table 1**. More than 93% of the CHI patients (n=57) fulfilled our alternative diagnostic criteria for CHI, i.e. hypoglycemia with a known disease-causing CHI mutation and/or CHI verified by histopathological analysis after pancreatic surgery. The last four samples were obtained from patients who had no identified mutations and were managed without pancreatic surgery. All patients with an alternative diagnosis of CHI were also identified with a clinical diagnosis of CHI. Three patients were diagnosed with Beckwith-Wiedemann syndrome and one was diagnosed with Kabuki syndrome. In the control group, data were available for 14 patients with a total of 15 samples with IKH.

**Table 1:** Characteristics of patients with CHI or IKH.

	CHI	IKH	*p* value
Patient numbers (%) Blood sample numbers (%)*	49 (77.8) 61 (80.3)	14 (22.2) 15 (19.7)	
Country of origin, no (%) Denmark Russia Ukraine Sweden Kazakhstan Belarus Portugal Serbia	5 (10.2) 13 (26.5) 10 (20.4) 8 (16.4) 5 (10.2) 3 (6.1) 3 (6.1) 2 (4.1)	13 (92.86) 1 (7.14)	<0.0001
Age, y, median (range)	0.45 (0.02-15)	2.8 (1.5–10)	<0.0001
Gender, no (%) Female Male	27 (55.1) 22 (44.9)	4 (28.6) 10 (71.4)	0.0580
Type, no (%) Transient Persistent	1 (2) 48 (98)		–
Genetic cause, no (%) K_ATP_-channel mutations Other genes No mutations found No data	37 (75.5) 2 (4.1) 9 (18.4) 1 (2)	14 (100)	
Histopathological analysis, no (%) Focal Diffuse Atypical (non-focal, non-diffuse) No data	26 (53.1) 12 (24.5) 3 (86.1) 8 (16.3)	14 (100)	<0.0001

CHI, congenital hyperinsulinism; IKH, idiopathic ketotic hypoglycemia.

*Eleven CHI patients had two and one patient had three samples eligible for analysis. One IKH patient had two samples eligible for analysis.

In comparison, the group of patients with CHI were significantly younger than the IKH control group, median 0.45 vs. 2.8 years, p*<* 0.0001.

### Fasting Tests and Diagnostic Performance of P-Insulin

Of the 61 included samples from CHI patients, four samples were included from patients with p-insulin >55 pmol/L during hypoglycemia despite ongoing treatment with octreotide. In IKH, two patients received inhalation corticosteroids in non-systemic doses for asthmatic bronchitis.

The median fasting duration was substantially shorter in the patients with CHI (0.5 vs. 12.5 h, p<0.0001), **Table 2**.

**Table 2:** Hypoglycemia sampling results in patients with CHI or IKH.

	CHI	IKH	*p-*value
P-glucose mmol/L, median (range)	2.6 (0.6–3.1); n=61	2.8 (2.3–3.1); n=15	0.0150
P-insulin pmol/L, median (range)	76.5 (17–644.3); n=61	1.5 (1.5–7.7); n=15	<0.0001
P-proinsulin pmol/L, median (range)	16 (3–200); n=58	1.5 (1.5–7)*; n=13	<0.0001
P-C-peptide pmol/L, median (range)	860 (225–5154); n=60	76 (32–109); n=15	<0.0001
Fast duration hours, median (range)	0.5 (0–10); n=42	12.5 (6–18); n=10	<0.0001
Glucose demand mg/kg/min., median (range)	11.6 (1–23.7); n=39	0	N/A
Concomitant medical treatment, no (%)	4** (6.4)	2*** (13.3)	N/A

*One outlier with p-proinsulin of 189 pmol/L not included.

**All four on octreotide treatment (patients included as p-insulin was >55 pmol/L).

***Both had inhalation corticosteroids in non-systemic dosage.

In CHI, the median (range) p-insulin concentration at p-glucose <3.2 mmol/L was 76.5 (17–644.3) pmol/L, compared to 1.5 pmol/L (1.5–7.7) pmol/L in patients diagnosed with IKH, p <0.0001. No samples from patients with CHI had insulin values below the normal reference range (15.1–147.1) pmol/L, while all samples from patients with IKH had p-insulin below normal range with 10 p-insulin values below LoQ (1.7 pmol/L).

**Figure 2:**
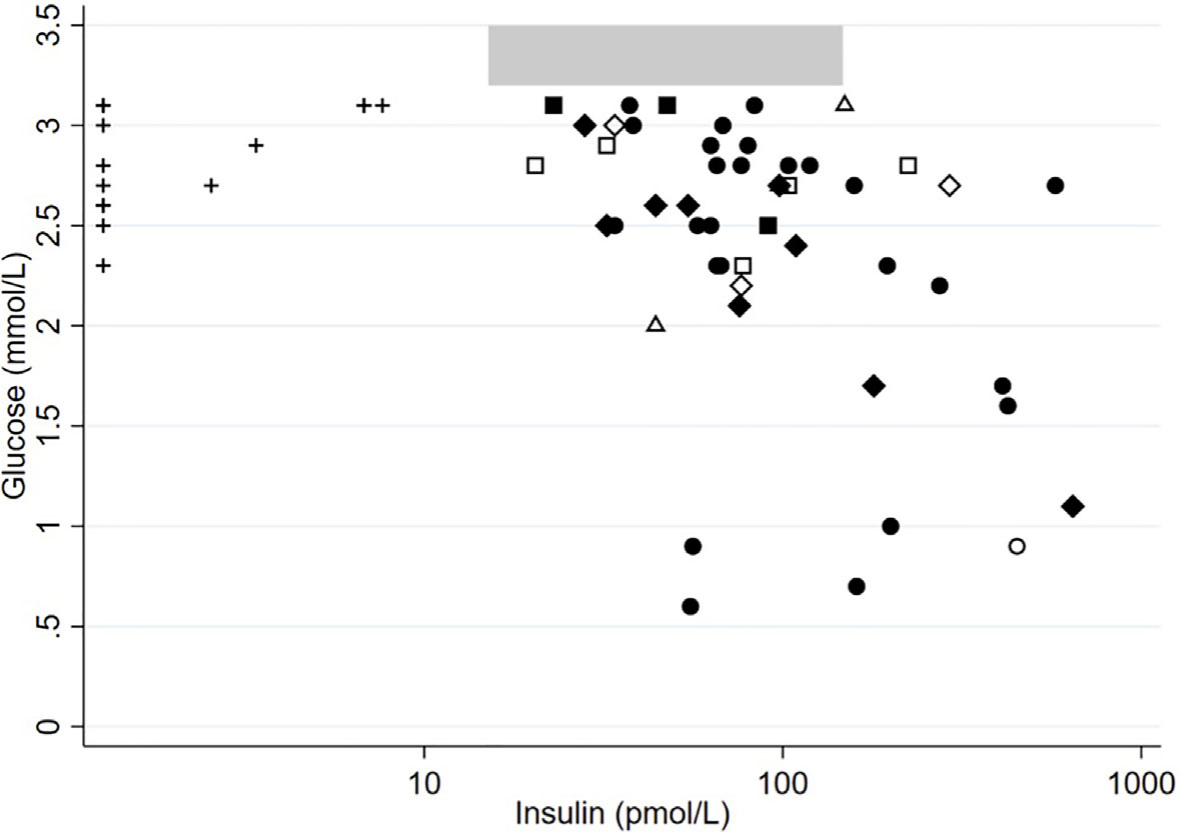
Two-way scatter-plot showing the association between p-insulin and p-glucose, split by IKH and subtypes of CHI. The X-axis is in logarithmic scale. Gray area indicates the normal insulin reference range 15.1–147.1 pmol/L for normal fasting glucose concentrations. +, IKH; ◆ and ◇, diffuse CHI; • and ○, focal CHI; Δ, atypical CHI; □, CHI with unknown histology. Closed symbols, K_ATP_-channel mutations. Open symbols, no K_ATP_ -channel mutations.

**Figure 2**** **shows the association between p-insulin and p-glucose for each sample. No inverse correlation between p-insulin and p-glucose was observed for the CHI samples. The histological subtypes of CHI showed no distinct distribution compared to each other. Moreover, samples from patients with and without K_ATP_-channel mutations had the same median (range) insulin-glucose ratio, 29.20 (7.40–585.73) vs. 35.49 (7.29–500.56) pmol/mmol, p= 0.84.

**Figure 3:**
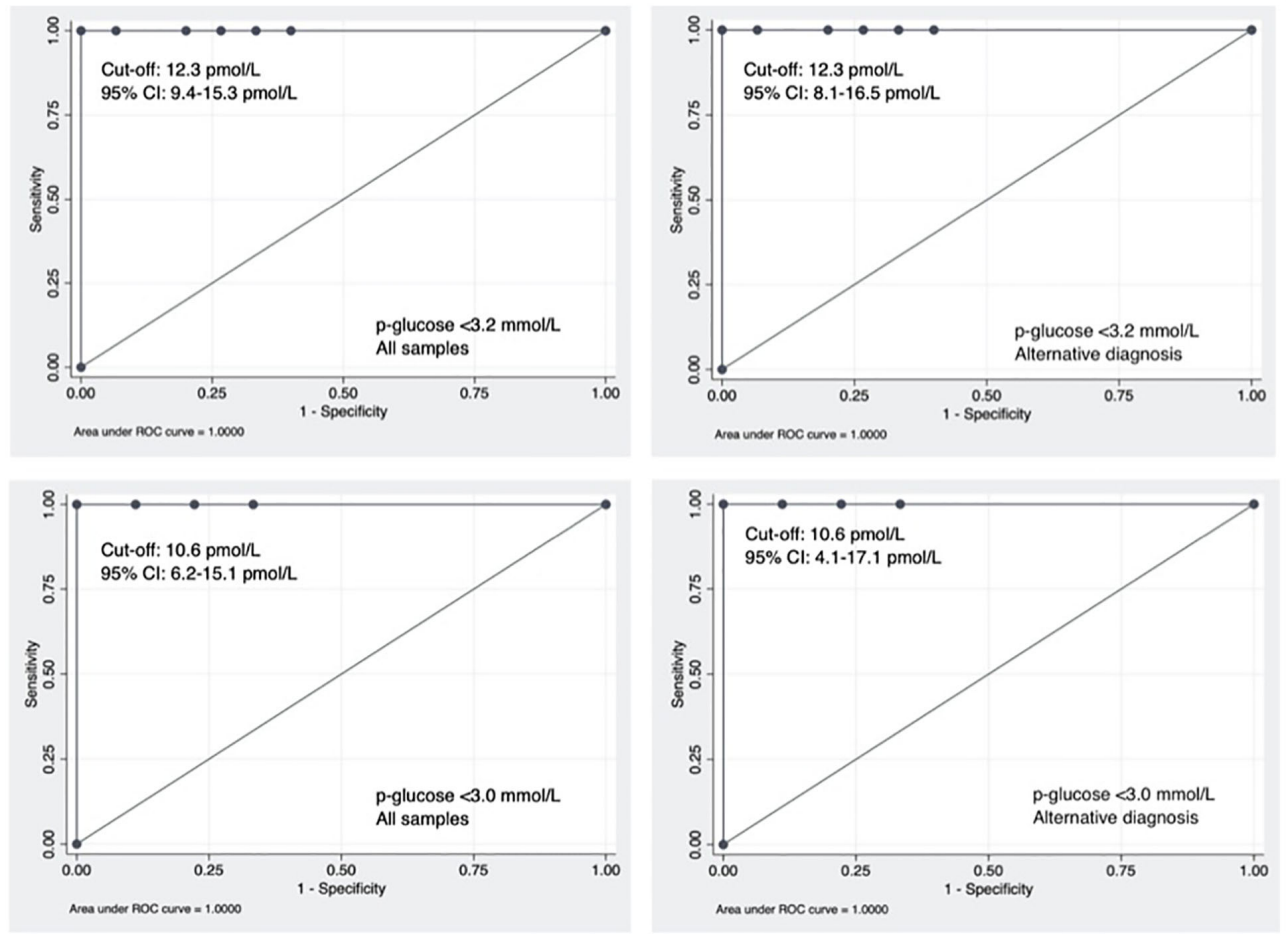
ROC-curves performed for the four predefined groups including cut-offs and 95% CI. Critical samples of p-insulin when simultaneous p-glucose was <3.2 mmol/L or <3.0 mmol/L. Alternative diagnosis of CHI was based on p-insulin free criteria.

The ROC curve AUC was 1.0 for the performance of p-insulin in diagnosing CHI, **Figure 3**. The optimal cut-off for p-insulin was 12.3 pmol/L (un-corrected 14.5 pmol/L) at p-glucose <3.2 mmol/L (n=61) with 100% sensitivity and specificity.

When only including samples with p-glucose <3.0 mmol/L (n=58), the optimal p-insulin cut-off was 10.6 pmol/L (uncorrected 12.5 pmol/L) with 100% sensitivity and specificity. There was no difference in the optimal cut-offs when including CHI patients by use of the alternative (p-insulin free) diagnosis. To control for oversampling, analyses with use of one sample from each patient was performed. Regardless of choosing the highest, or the first, p-insulin from each patient, the optimal p-insulin cut-off was exactly the same as in the primary analysis.

### P-Proinsulin, C-Peptide, and Blood Ketones

P-proinsulin and p-C-peptide were clearly elevated in the samples from patients with CHI compared to the patients with IKH and blood ketones were suppressed in all patients with CHI and elevated in all patients with IKH, **Table 2**. The highest ketone concentration was 0.6 mmol/L in CHI patients with a clinical or alternative diagnosis of CHI.

### Insulin–Glucose Ratios

The median (range) insulin-glucose ratio in samples from patients with CHI (n=61) was 33.4 (76.5–586) pmol/mmol, **Table 3**. The PPV for the diagnosis of CHI by a ratio >32.2 pmol/mmol was 0.508 only. The amended insulin-glucose ratio was 78.6 (16.4–646) pmol/mmol with a PPV for the diagnosis of CHI by a ratio >53.6 pmol/mmol of 0.647. By use of corrected insulin conversion factor, the diagnostic positive predictive value for the ratios were 0.558, and 0.706, respectively.

**Table 3:** Insulin–glucose ratios in patients with CHI or IKH.

	CHI	IKH
Simple insulin-glucose ratio pmol/mmol, median (range) Identified correctly, no/no (%)* Un-corrected Corrected (-15%)	33.4 (6.5–585.7) 31/61 (50.8) 34/61 (55.8)	0.5 (0.4–2.5) 15/15 (100) 15/15 (100)
Amended insulin-glucose ratio, pmol/mmol, median (range) Identified correctly, no/no (%)* Un-corrected Corrected (-15%)	78.6 (16.4–646) 33/51 (64.7) 36/51 (70.6)	1.6 (0.9–5.5) 15/15 (100) 15/15 (100)

*Based on normative references, here given without and with corrected conversion factor for conversion of p-insulin from mU/L to pmol/L.

The median (range) insulin-glucose ratio for patients with IKH was 0.5 (0.4–2.5) pmol/mmol; amended insulin-glucose ratio 1.6 (0.9–5.5) pmol/mmol. All patients with IKH were therefore identified as having normal ratios, also when using the conversion factor corrected cut-off.

## Discussion

In our setting, the use of a sensitive insulin assay gave a diagnostic performance of the p-insulin concentration during hypoglycemia with AUC=1.0 in all ROC-curve analyses and 100% sensitivity and specificity in diagnosing CHI.

Lord et al. (10) and Thorton et al. (14) defined p-insulin cut-off for the diagnosis of CHI as detectable p-insulin during hypoglycemia with need of supporting evidence from additional diagnostic criteria. Ferrara et al. (15) found that <20% of CHI-patients had undetectable (<2-3 mU/L) insulin concentrations during hypoglycemia and stated that supporting biomarkers are needed.

Our study showed that the optimal cut-off is below the normal range and below, or at, the threshold of detecting insulin in most assays. With the sensitive assay, the diagnosis of CHI could be made exclusively based on p-insulin above cut-off during hypoglycemia in our data set.

If valid in other settings, the use of a sensitive insulin assay may have an impact on currently recommended diagnostic procedures which rely on a combination of p-insulin, supportive biomarkers and inappropriately large glycemic response to i.m. glucagon.

With the sensitive assay, we were able to investigate p-insulin even at values below the normal range. None of our CHI patients had p-insulin below the lower normal value of 15.1 pmol/L (2.52 mU/L; or 2.17 mU/L without correction factor). The obtainment of this precise cut-off could be due to our strict exclusion criteria of ongoing anti-insulin treatment, the strict demand of simultaneously drawn blood for p-glucose determined by gold standard methods instead of point-of-care devices, or the commonly occurring selection bias with a high representation of severely affected patients in tertiary, national or international referral centers. The external validity of our findings should be investigated in other settings before the diagnostic approach to CHI may be changed.

In routine settings, the conversion factor from mU/L to pmol/L is 6.945 for insulin. Larsen et al. (23) showed that the Cobas e411 assay uses a conversion factor that disagrees with the biological activity of insulin with <15%, concluding that the correct conversion factor is 6.0. Our ROC AUCs for p-insulin cut-offs and conclusion on insulin-glucose ratios remained unchanged whether or not a corrected conversion factor was used. Clinicians should be aware of the fact that routine insulin concentrations reported in pmol/L should be corrected.

Measurement of C-peptide and proinsulin did not give additional information in our setting, but may still be considered in patients where differentiation between insulin secretion and insulin production (a high proinsulin: insulin ratio) is needed, or when exogeneous insulin administration is suspected (high insulin, low C-peptide and proinsulin) (14).

Cryer et al. (26) found that simple insulin-glucose ratios had no diagnostic utility in patients with insulinoma. In contrast, amended insulin-glucose ratios had sensitivity and specificity of 98% in insulinoma (19). In our setting, both the simple and amended insulin-glucose ratio had a low diagnostic accuracy and should be considered inadequate as diagnostic tools in CHI.

Blood ketones did not add additional information in the diagnosis of our CHI patients, where the highest ketone concentration was 0.6 mmol/L. Rare CHI patients have been reported with elevated beta-hydroxybutyrate; some without further explanatory details; some with *PGM1*-mutations and fasting ketotic hypoglycemia but post-prandial hypoketotic CHI; and others with ketotic hypoglycemia during ongoing diazoxide treatment (27–29).

Our study included 3½ times more patients with CHI than IKH. This does not reflect in prevalence of the two diseases, as the majority (90%) of CHI patients were referred from other countries to our international CHI center. Moreover, not all IKH patients at our center were included due to missing or unqualified p-insulin samples.

## Strengths and Limitations

Strengths of our study included the sensitive insulin assay, the strict inclusion and exclusion criteria with standardized fasting tests without medication, the use of gold standard p-glucose measurement and p-insulin from simultaneously drawn blood samples, and the robustness of our findings when using different glucose thresholds, different CHI diagnostic criteria, and different conversion factors from mU/L to pmol/L for p-insulin.

Limitations included the retrospective and single-center design, the relatively few IKH control patients, the well-known age difference between CHI and IKH patients, and potential selection bias due to a high percentage of referred patients from other countries.

## Conclusion

The sensitive insulin assay performed excellent in diagnosing CHI with a ROC AUC of 1.0. The use of a p-insulin cut-off of 10.6–12.3 pmol/L during a diagnostic hypoglycemia test may establish the diagnosis of CHI without further diagnostic testing. The external validity of our findings should be tested in other settings.

## Data Availability Statement

The raw data supporting the conclusions of this article will be made available by the authors, without undue reservation.

## Ethics Statement

The Region of Southern Denmark, Protocol no. 19/7744, and the Danish Patient Safety Authority, Protocol no. 3-3013-2884/1, approved the study protocol.

## Author Contributions

HC conceived the idea. JS did the data collection and performed the analyses. HC and JS wrote the manuscript. All authors provided critical feedback and helped shape the research, analysis, and manuscript. All authors contributed to the article and approved the submitted version.

## Conflict of Interest

The authors declare that the research was conducted in the absence of any commercial or financial relationships that could be construed as a potential conflict of interest.
